# Supplementary material for: Metallic Stent Mesh Coated with Silver Nanoparticles Suppresses Stent-Induced Tissue Hyperplasia and Biliary Sludge in the Rabbit Extrahepatic Bile Duct
Source: Pharmaceutics. 2020 Jun 17;12(6):563. doi: 10.3390/pharmaceutics12060563 (PMC7356520; doi:10.3390/pharmaceutics12060563)
Supplement: Supplementary file 1 [file pharmaceutics-12-00563-s001.pdf]

pharmaceutics

Supplementary Materials for

Metallic Stent Mesh Coated with Silver Nanoparticles Suppresses Stent-Induced Tissue Hyperplasia and Biliary Sludge in the Rabbit Extrahepatic Bile Duct

Table S1. Characterization of AgNPs-coated SEMS.

Groups	Feed amount		EDS analysis	
	PDA [a] (mg)	$AgNO_3$ [b] (mg)	Carbon [c] (%)	Ag [d] (%)
**A**	0	0	7	0
**B**	15	45	28	22
**C**	15	90	24	34
**D**	15	180	20	55

[a] Weight of feed dopamine hydrochloride per SEMS in 15 mL of 5 mM Tris buffer; [b] Weight of feed $AgNO_3$ per SEMS in 15 mL of DW; [c] Percent of carbon in the total area, as determined by EDS mapping; [d] Percent of Ag in the total area, as determined by EDS mapping; Note. DW; deionized water, PDA; dopamine hydrochloride, SEMS; self-expandable metallic stent.

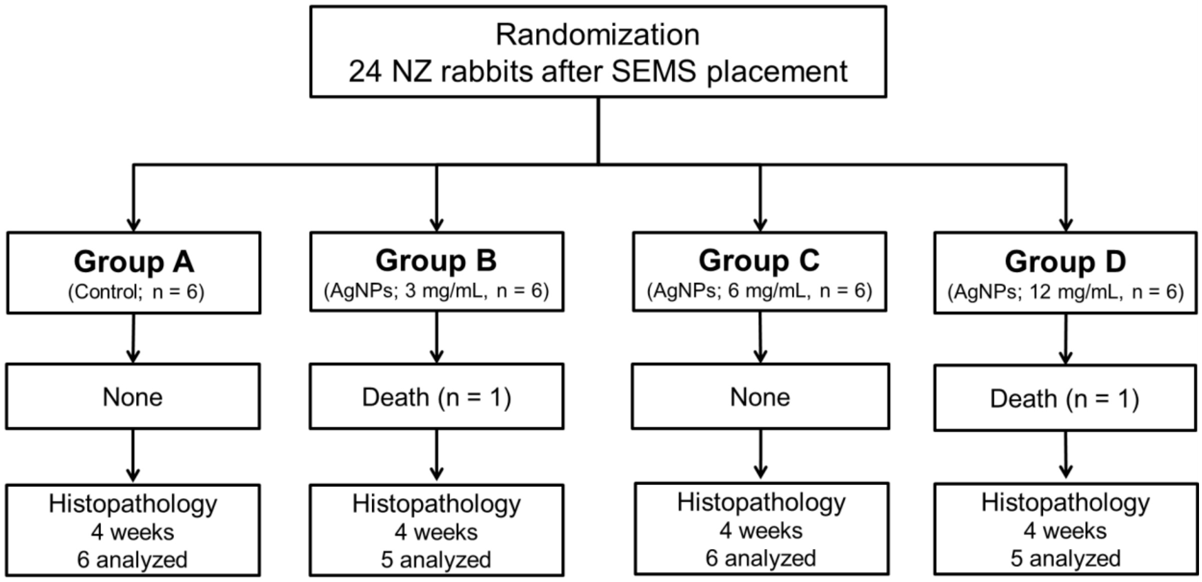

Figure S1. Flow diagram and study design showing the randomization process and follow-up. Note: NZ, New Zealand; SEMS, self-expandable metallic stent; AgNPs, Ag nanoparticles.

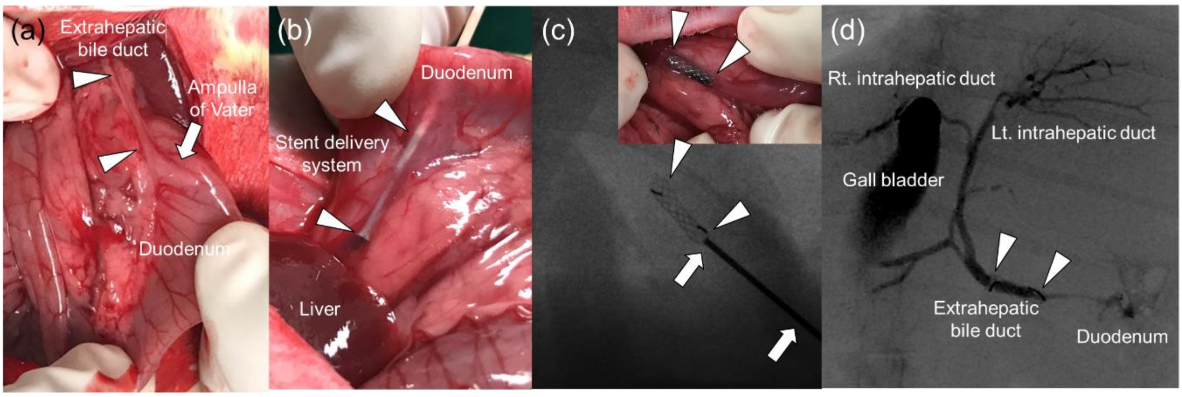

Figure S2. Photographic and radiographic images showing the technical steps involved in stent placement in the rabbit extrahepatic bile duct. (**a**) A photograph identifying the ampulla of Vater (arrow), the extrahepatic bile duct (arrowheads), and the duodenum. (**b**) Photograph obtained during stent placement showing a compressed stent (arrowheads), which was loaded in the angiocatheter and positioned in the extrahepatic bile duct. (**c**) Photograph and radiograph showing a placed stent (arrowheads) in the rabbit extrahepatic bile duct and a pusher rod (arrows). (**d**) Post-procedural cholangiography showing good passage of contrast medium through the stent (arrowheads) without any procedure-related complications.

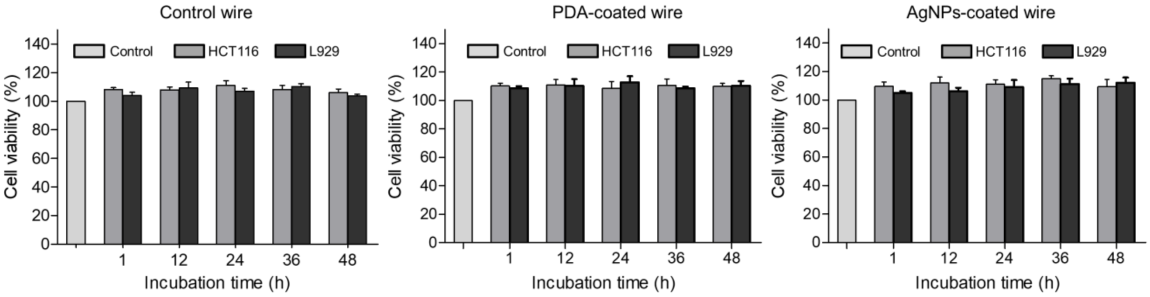

Figure S3. Cytotoxicity analysis of control nitinol wire, PDA-coated wire, and AgNPs-coated wire after incubation with L929 and 293 cells for different periods of time.
